# Sociodemographic Characteristics of Women Able to Obtain Medication Abortion Before and After Ohio's Law Requiring Use of the Food and Drug Administration Protocol

**DOI:** 10.1089/heq.2018.0002

**Published:** 2018-07-01

**Authors:** Ushma D. Upadhyay, Nicole E. Johns, Alice F. Cartwright, Tanya E. Franklin

**Affiliations:** ^1^ Advancing New Standards in Reproductive Health (ANSIRH), Bixby Center for Global Reproductive Health, Department of Obstetrics, Gynecology, and Reproductive Sciences, University of California, San Francisco, Oakland, California.; ^2^ Department of Obstetrics, Gynecology, and Women's Health, University of Louisville School of Medicine, Louisville, Kentucky.

**Keywords:** abortion, health policy, medication abortion, Ohio, sociodemographic characteristics

## Abstract

**Purpose:** In 2011, a law went into effect in Ohio that regulates how abortion care providers can offer medication abortion to their patients. We sought to evaluate changes in sociodemographic characteristics of Ohio medication abortion patients before and after the implementation of this law.

**Methods:** We used a retrospective cohort design, comparing characteristics of women obtaining a medication abortion at four abortion facilities before and after the law. We used chart data from January 2010 to January 2011 and February 2011 to October 2014. For any significant changes in sociodemographics found before and after the law, we used stratified cross-tabulations to disentangle whether they were likely related to the restricted gestational limit imposed by the law (lowered from 9 to 7 weeks gestation), or whether they were likely related to other burdens brought on by the law, such as increased costs and visits.

**Results:** Women obtaining a medication abortion after the law were more likely to be older (*p*=0.01), have higher levels of education (*p*<0.001), be of white race (*p*<0.001), have private insurance (*p*=0.001), have no children (*p*=0.002), and reside in a higher income zip code (*p*=0.03). Both the reduced gestational limit and the increased costs and visits likely contributed to declines among black women and women with lower levels of education. The reduced gestational limit for medication abortion likely contributed to a decline among younger women and Medicaid recipient groups. The increased costs and visits imposed by the law likely contributed to the decline in medication abortion among women with no insurance and women with children.

**Conclusion:** The lower gestational limit, higher cost, and time and travel burdens exacted by Ohio's medication abortion law were associated with disproportionate reductions in medication abortion among the most disadvantaged groups. The law was associated with reduced access among women who were younger, of black race, less educated, and in lower socioeconomic groups.

## Introduction

Medication abortion is a nonsurgical abortion in which two medications, mifepristone (brand name: Mifeprex) and misoprostol, are taken 24–48 h apart to induce an abortion. The mifepristone is an antiprogestin that stops the pregnancy from continuing, and the misoprostol causes the cervix to soften and the uterus to contract, resulting in the expulsion of the contents of the uterus. The physical process of having a medication abortion is similar to a miscarriage. While abortion with pills is sometimes called a medical abortion, and was previously called RU-486, the term medication abortion most accurately represents the use of drug-based methods that can terminate pregnancy.^1^

The method was developed in France and approved there in 1988.^2^ It underwent a 54-month politically charged review in the United States, and was ultimately approved by the Food and Drug Administration (FDA) in 2000.^2,3^ The 2000 FDA protocol recommended its use only up to 7 weeks of pregnancy, involving a higher dose (600 mg) of the more expensive drug, mifepristone, and a lower dose (400 mcg) of the cheaper drug, misoprostol. For those who wanted a medication abortion, the 2000 FDA protocol required patients to visit a provider three times: first to take the mifepristone pills, second to take the misoprostol pills, and third for a follow-up visit to ensure the abortion was complete. This protocol is 92% effective only up to 7 weeks gestation.^4^

In the subsequent years after the initial registration of mifepristone in 2000, research and clinical practice demonstrated that an updated evidence-based protocol was more convenient, and more effective than the originally approved FDA protocol, with an effectiveness rate of 95–99% when administered up to 9 weeks gestation.^5–7^ The evidence-based protocol permitted use up to 9 weeks of pregnancy, involving a lower dose (200 mg) of the expensive mifepristone and a higher dose of cheaper misoprostol (800 mcg). But probably the largest change from a patient perspective is that it eliminated a visit by not requiring patients to return to take the second set of pills, the misoprostol, in front of the provider. Research had demonstrated that women could be safely entrusted to take them at home^8,9^ according to instructions, although a follow-up visit was still usually required. The 2010 National Abortion Federation guidelines recommended the evidence-based protocol.^10^ By 2011, most providers in the United States were no longer using the FDA protocol, and instead were using the evidence-based protocol.^11^

However, in 2011 a law went into effect in Ohio that regulated how abortion care providers could offer medication abortion to their patients. At the time, this law required clinicians to provide medication abortion according to the Food and Drug Administration protocol, as it was originally approved in 2000 which was still the official label (Table 1). Since in Ohio, all abortion patients must have a mandated information visit, followed by a 24-h waiting period before they can obtain the mifepristone, providing care according to the 2000 protocol increased the total number of required visits to four.

**Table 1.** **Protocol Comparison**

	Evidence-based regimen	Original FDA-approved regimen (as approved in 2000)
Dates in use in study data	January 2010–January 2011	February 2011–March 2016
Maximum days gestation	9 weeks from LMP^a^	7 weeks from LMP
Mifepristone dose	200 mg orally in office	600 mg orally in office
Misoprostol dose	800 mcg vaginally or buccally (four tablets)	400 mcg orally (two tablets)
Misoprostol timing	6–72 h after mifepristone	48 h after mifepristone
Misoprostol location	Home	Provider's office
Follow-up visit	5–14 days after mifepristone	14 days after mifepristone
Cost	Lower	Higher^b^
Minimum number of office visits (including Ohio's required information visit)	3	4
Efficacy rate	95–99% up to 9 weeks gestation	92% up to 7 weeks gestation

Adapted from Reproductive Health Access Project^44^ and American College of Obstetricians and Gynecologists.^6^

^a^ In 2016, the FDA approved use up to 10 weeks from LMP.

^b^ The original FDA protocol is more costly because it requires three times the dose of mifepristone which is a more costly drug than misoprostol.

FDA, Food and Drug Administration; LMP, last menstrual period.

In March 2016, the FDA updated the recommended protocol for Mifeprex to bring it in line with the clinical evidence base. These updates included expanding eligibility from 7 to 10 weeks gestation. It reduced the number of clinical office visits required from three visits to one; women could now obtain both medications in a single visit and take the first pills (mifepristone) to start their abortion when they wanted instead of at the doctor's office when they were able to get an appointment. The protocol no longer required patients to have an in-office follow-up appointment, allowing for alternatives to the follow-up visit.^12^ Thus, the new March 2016 protocol required only one visit for a medication abortion (but still two in Ohio with the mandated information visit).

**FIG. 1.**
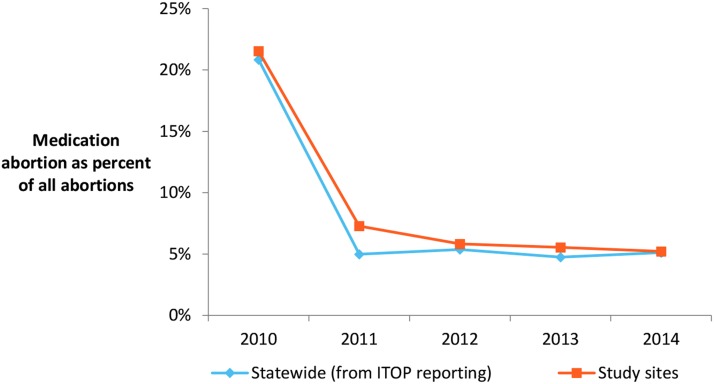
Medication abortion as a percentage of all abortions in Ohio 2010–2014, study clinic and overall state data. State data from Ohio Department of Health ITOP statistics. ITOP, Induced Termination of Pregnancy.

Our previous research has demonstrated that after the 2011 Ohio law was enacted, women obtaining medication abortions were three times as likely to require additional treatments to complete their abortions compared with women before the law went into effect.^13^ We also found that the proportion of medication abortion patients at four facilities in the state declined dramatically, from 22% of all abortions in 2010 down to 5% in 2014. There were no other changes in the provision of care or insurance acceptance during the same period at these facilities. State-level data confirm this finding: medication abortion fell significantly from 21% of all abortions in the state in 2010 to 5% of abortions in 2011 (*p*<0.001) where it remained through 2014 (Fig. 1).^14–18^ In addition, other statewide analyses of mifepristone use between the years 2004–2014 in four states with large populations of women of reproductive age (including Ohio) corroborate massive declines in mifepristone use when the laws went into effect in Ohio and Texas, compared with steady and continued increases in mifepristone use in two states that did not have any restrictions on medication abortion (California and New York).^19^ Further national data from the same period on nonhospital abortions demonstrate a steady increase in the proportion of medication abortions from 17% in 2008 to 24% in 2011 to 31% in 2014.^20,21^

Having the option of medication abortion is important to many women seeking abortion. Some women prefer medication abortion for several reasons: so they can be at home for the abortion, avoid aspiration, surgery, and/or anesthesia, and because they believe the method to be less invasive, painful, or frightening, or more natural or safer.^22,23^ When women are offered a choice between medication abortion and aspiration, they find the method they personally choose for themselves to be highly acceptable.^24,25^ However, there is no evidence demonstrating that sociodemographic characteristics affect patient preferences between medication abortion and aspiration abortion.^22,26^ Research has consistently shown that medication abortion is highly acceptable to women from diverse racial/ethnic groups, education levels, and income levels.^22,26–29^

Due to factors intersecting at the individual, societal, and health system levels,^30^ women of color and low-income women are disproportionately represented among abortion patients relative to the general population in the United States. Black and Hispanic women are overrepresented, and three-quarters of abortion patients are low income (<200% of the federal poverty level).^31,32^ One of the reasons for high relative rates of abortion is that rates of unintended pregnancies are higher among populations of women of color, low-income women, and women with lower educational attainment.^33,34^ Differences in rates of unintended pregnancy are related to disparities in rates of contraceptive use. Systematic barriers to contraceptive access such as lack of health insurance coverage or high out-of-pocket costs for certain methods can make contraception out of reach for many women. In addition, concerns about safety and fears of coercion related to hormonal contraception and sterilization (more effective methods)^35^ stem from the history of discriminatory practices directed at controlling and devaluing the fertility of low-income women and women of color.^36–39^

In our previous research, we found that the sociodemographic characteristics of women who had medication abortions under 7 weeks gestation changed significantly following the implementation of the FDA protocol requirement.^13^ However, that analysis was unable to determine whether the changes in sociodemographic characteristics were only because it excluded women 7–9 weeks gestation, or whether the barriers imposed by the law systematically prevented certain groups of women from having a medication abortion. In this analysis, we look at all women who were able to obtain a medication abortion before and after the law, regardless of weeks gestation, to determine the impact of the law by sociodemographic characteristics.

## Materials and Methods

We abstracted medical chart data at four abortion-providing facilities in Ohio for all medication abortions from 1 year prior to the law's implementation (January 2010–January 2011) to 3 years postimplementation (February 2011–October 2014). We chose to abstract 1 year of prelaw data and 3 years of postlaw data because after the law went into effect, the number of medication abortions dropped to such a degree that we needed to collect 3 years of data to reach the *a priori* calculated sample size required to achieve sufficient statistical power. The Institutional Review Board of the University of California, San Francisco, granted ethical approval for this study. The data collection methodology has been previously described.^13^ Abstraction occurred between September 2014 and April 2015.

### Measures

Sociodemographic measures abstracted from patient charts included age, highest level of education, race/ethnicity, insurance status, zip code, height, weight, and previous births. (In Ohio, Medicaid cannot be used to pay for abortion, and thus insurance status did not necessarily reflect how the patient paid for the abortion; we use it as a proxy for socioeconomic status.) Distance traveled to abortion care was calculated based on home zip code (the most detailed location information available) to facility using the “traveltime3” STATA module, which utilizes a Google Maps application programming interface to calculate driving distance. Clinical information beyond the consent visit was not abstracted for women at gestations 7–9 weeks in the prelaw period, so we used gestation at the consent visit rather than gestation at the mifepristone visit. Previous births were used as an imperfect proxy for having children. When missing, the number of previous births was computed based on the number of previous vaginal births, number of previous cesarean sections, number of previous pregnancies, number of previous abortions, and number of previous miscarriages. Zip-code-based household income quintile information was obtained from 2010 census data via the Michigan Population Studies Center.^40^ All other data were analyzed as they appeared in the chart.

### Data analysis

We performed the analysis in two parts: First, we compared characteristics of the overall study population obtaining a medication abortion at the four study sites before and after the law. We compared the distributions of sociodemographic characteristics of women able to obtain a medication abortion at any gestation by first examining results from overall chi-square tests. If the overall chi-square test was statistically significant, at *p*<0.05, we conducted group-wise *t*-tests to learn which group differences were statistically significant. In the text, we report the overall *p*-values for ordinal categorical variables and individual *p*-values for nominal categorical variables when statistically significant at *p*<0.05.

Second, we sought to further disentangle whether any changes in sociodemographics found were likely related to the restricted gestational limit imposed by the law (lowered from 9 to 7 weeks gestation), or whether they were likely related to other factors impacted by the law, such as increased costs and visits. To do so, we repeated chi-square tests of distributions stratified by gestation, comparing those patients at <7 weeks gestation prelaw with those patients 7–9 weeks gestation prelaw (to examine whether differences were potentially associated with changing gestational limits) and comparing those patients at <7 weeks gestation prelaw with those patients <7 weeks gestation postlaw (to examine differences potentially associated with law-related factors other than gestational limits). We report chi-square and *t*-test results similarly to the analysis above. All statistical analyses were conducted using Stata version 14.0.

## Results

The decline in medication abortion was not evenly distributed across sociodemographic groups; we found significant differences in the characteristics of women obtaining medication abortion before and after the law (Table 2). As a direct result of the law which lowered the legal gestational limit for medication abortion from 9 to 7 weeks, women in the postlaw period had their medication abortions at significantly earlier gestations (*p*<0.001). In the prelaw period, about one-third of the medication abortions were occurring between 7 and 9 weeks gestation. This dropped to 0% in the postlaw period as a result of the law.

**Table 2.** **Characteristics of the Population Obtaining a Medication Abortion Among Patients from Four Abortion-Providing Facilities in Ohio, 2010–2014**

	Prelaw	Postlaw	Total	Significance prelaw vs. postlaw^a^
*N*, numbers	2169	1627	3796	
Age, %				0.01
<20	15.4	12.0	13.9	0.003
20–24	35.8	34.4	35.2	N.S.
25–29	23.9	26.0	24.8	N.S.
30–39	21.9	24.2	22.9	N.S.
40+	3.0	3.4	3.2	N.S.
Highest level of education, %				<0.001
Less than high school degree	11.9	7.6	10.0	<0.001
High school diploma or GED	40.8	35.5	38.5	0.001
Associates degree/some college	25.1	29.4	27.0	0.004
Bachelors degree or higher	13.6	23.2	17.7	<0.001
Not in chart	8.7	4.3	6.8	<0.001
Race/ethnicity, %				<0.001
White	65.2	71.3	67.8	<0.001
Black	23.5	15.7	20.2	<0.001
Latina	3.8	4.6	4.1	N.S.
Asian/Pacific Islander	3.1	3.9	3.5	N.S.
Other/not in chart	4.4	4.5	4.4	N.S.
Insurance, %				<0.001
Private	23.2	33.7	27.7	<0.001
Medicaid/Medicare	19.9	17.1	18.7	0.03
None	28.7	24.4	26.8	0.003
Not in chart	28.2	24.8	26.7	0.02
Distance traveled for care, %				N.S.
<50 miles	86.5	85.7	86.2	
50+ miles	12.1	13.4	12.6	
Not in chart	1.4	0.9	1.2	
Urban/rural, %				N.S.
Urban	93.5	93.4	93.5	
Rural	5.3	5.9	5.6	
Not in chart	1.2	0.7	1.0	
BMI category, %				N.S.
Underweight (<18.5)	3.5	3.6	3.5	
Healthy weight (18.5–25)	45.8	51.6	48.3	
Overweight (25–30)	24.7	23.5	24.2	
Obese (30–35)	9.1	9.1	9.1	
Morbidly obese (35+)	7.5	7.0	7.3	
Not in chart	9.4	5.2	7.6	
Gestation at consent, %				<0.001
Up to 4 weeks 6 days	10.7	19.4	14.5	<0.001
5 weeks 0 day to 5 weeks 6 days	22.1	45.4	32.1	<0.001
6 weeks 0 day to 7 weeks 0 day	33.0	35.2	33.9	N.S.
7 weeks 1 day to 9 weeks 0 day	31.6	0.0	18.1	<0.001
Not in chart	2.6	0.0	1.5	<0.001
Previous births, %				0.002
0	49.5	54.7	51.7	0.002
1 or more	49.9	44.9	47.8	0.002
Not in chart	0.6	0.4	0.5	N.S.
Zip-code-based national household income quintile, %				0.03
0–20	1.9	1.2	1.6	N.S.
20–40	18.3	17.4	17.9	N.S.
40–60	54.4	52.2	53.4	N.S.
60–80	23.1	26.8	24.7	0.01
80–100	0.9	1.4	1.1	N.S.
Not in chart	1.5	1.0	1.3	N.S.

^a^ *p*-Values for overall characteristic chi-square tests based on in-chart data only.

BMI, body mass index; GED, general educational development; N.S., pre- and postlaw differences not statistically significant.

The population obtaining a medication abortion in the postlaw period was significantly more likely to be older (*p*=0.01), have higher levels of education (*p*<0.001), be of white race (*p*<0.001), have private insurance (*p*<0.001), and have no children (*p*=0.002) than women obtaining medication abortion before the law went into effect. In addition, in the post law period this population was significantly less likely to be of black race (*p*<0.001), and less likely to have Medicaid (*p*=0.03) or no health insurance at all (*p*=0.003) compared with women in the prelaw period. The distribution by body mass index did not change significantly after the law. Residence-related measures did not show as much change; there was no significant change in distance traveled or urban/rural residence before and after the law, but women obtaining medication abortion after the law were less likely to reside in a low-income zip code (one of the bottom two quintiles of household wealth, overall *p*=0.03).

When we compared populations at <7 weeks prelaw and 7–9 weeks prelaw as well as populations <7 weeks pre- and postlaw, a few characteristics were significantly different for both comparisons, namely race/ethnicity and education (Table 3). This suggests that changes in the distributions of women by race/ethnicity and education were potentially driven by changes in the gestational limit and by other factors related to the law. In the prelaw period, black women were more heavily represented in the population obtaining abortions at 7–9 weeks (27%) than those obtaining abortions at <7 weeks (21%, *p*<0.001), so the gestational limit change would be expected to affect the number of black women obtaining medication abortions. In addition, limiting the comparison between pre- and postlaw distributions to those obtaining abortions at <7 weeks, there is still a significantly lower representation of black women in the postlaw period (16%) than the prelaw period (21%, *p*<0.001). Controlling for gestation by looking at women only <7 weeks, there appears to be an association between other factors related to the law and black women's ability to obtain an abortion.

**Table 3.** **Characteristics of Populations Obtaining Medication Abortion at <7 Weeks Gestation Prelaw, 7–9 Weeks Gestation Prelaw, and <7 Weeks Gestation Postlaw at Four Abortion-Providing Facilities in Ohio, 2010–2014**

	Comparison of prelaw populations by gestation (effect of law likely due to lowered gestational limit)			Comparison of populations at <7 weeks gestation by time period (effect of law due to other burdens imposed by law)		
	Prelaw <7 weeks	Prelaw 7–9 weeks	Significance: <7 weeks vs. 7–9 weeks prelaw^a^	Prelaw <7 weeks	Postlaw <7 weeks	Significance: <7 weeks prelaw vs. <7 weeks postlaw^a^
*N*, numbers	1156	1013		1156	1627	
Age, %			<0.001			N.S.
<20	14.3	16.7	N.S.	14.3	12.0	
20–24	33.3	38.7	0.009	33.3	34.4	
25–29	23.7	24.2	N.S.	23.7	26.0	
30–39	25.2	18.2	<0.001	25.2	24.2	
40+	3.5	2.3	N.S.	3.5	3.4	
Highest level of education, %			<0.001			<0.001
Less than high school degree	10.1	13.9	0.006	10.1	7.6	0.02
High school diploma or GED	38.3	43.5	0.01	38.3	35.5	N.S.
Associates degree/some college	27.7	22.2	0.003	27.7	29.4	N.S.
Bachelor's degree or higher	14.9	12.0	N.S.	14.9	23.2	<0.001
Not in chart	9.0	8.3	N.S.	9.0	4.3	<0.001
Race/ethnicity, %			0.001			0.001
White	68.2	61.9	0.002	68.2	71.3	N.S.
Black	20.7	26.8	<0.001	20.7	15.7	<0.001
Latina	3.3	4.3	N.S.	3.3	4.6	N.S.
Asian/Pacific Islander	3.8	2.3	0.04	3.8	3.9	N.S.
Other/not in chart	4.1	4.7	N.S.	4.1	4.5	N.S.
Insurance, %	<0.001			<0.001		
Private	26.6	19.4	<0.001	26.6	33.7	<0.001
Medicaid/Medicare	17.2	23.0	<0.001	17.2	17.1	N.S.
None	30.1	27.1	N.S.	30.1	24.4	<0.001
Not in chart	26.0	30.6	0.02	26.0	24.8	N.S.
Distance traveled for care, %			N.S.			N.S.
<50 miles	86.7	86.4		86.7	85.7	
50+ miles	12.1	12.0		12.1	13.4	
Not in chart	1.2	1.6		1.2	0.9	
Urban/rural, %			N.S.			N.S.
Urban	94.0	92.9		94.0	93.4	
Rural	4.9	5.7		4.9	5.9	
Not in chart	1.0	1.4		1.0	0.7	
BMI category, %			N.S.			N.S.
Underweight (<18.5)	3.4	3.6		3.4	3.6	
Healthy weight (18.5–25)	47.5	43.9		47.5	51.6	
Overweight (25–30)	25.9	23.4		25.9	23.5	
Obese (30–35)	8.7	9.6		8.7	9.1	
Morbidly obese (35+)	8.0	7.0		8.0	7.0	
Not in chart	6.7	12.5		6.7	5.2	
Previous births, %			N.S.	0.005		0.005
0	49.2	49.9		49.2	54.7	0.004
1 or more	50.3	49.6		50.3	44.9	0.006
Not in chart	0.5	0.6		0.5	0.4	N.S.
Zip-code-based national household income quintile, %			N.S.			N.S.
0–20	1.3	1.7		1.3	1.2	
20–40	1.5	2.4		1.5	17.4	
40–60	17.0	19.7		17.0	52.2	
60–80	54.2	54.6		54.2	26.8	
80–100	25.1	20.9		25.1	1.4	
Not in chart	1.0	0.7		1.0	1.0	

^a^ *p*-Values for overall characteristic chi-square tests based on in-chart data only.

Similarly, in the prelaw period, women with lower levels of education (less than high school and high school/general educational development) were more heavily represented in the population obtaining abortions at 7–9 weeks than those obtaining abortions at <7 weeks (overall *p*<0.001). Comparing pre- and postlaw distributions of women obtaining abortions at <7 weeks, we find significantly lower representation of women with less than high school in the postlaw period (overall *p*<0.001). These results suggest that this change was associated with both the change in gestational limit and other factors imposed by the law, such as increased visits and costs.

The age distribution was significantly different between women at <7 weeks and those at 7–9 weeks gestation in the prelaw period but not for women at gestations <7 weeks before and after the law. Similarly, in the prelaw period women with Medicaid are overrepresented in the population obtaining abortions at 7–9 weeks (23%) compared with those <7 weeks (17%), but there was no difference among women <7 weeks pre- and postlaw. These comparisons suggest that the shift toward older age and reductions in Medicaid recipients after the law were potentially driven by the restriction to earlier gestations under the law.

In contrast, we see that there is no significant difference in the percentage of patients with no health insurance <7 weeks and 7–9 weeks before the law; however, there was a statistically significant drop in the proportion of women with no health insurance <7 weeks after the law (30–24%, *p*<0.001). Similarly, there was no difference in the proportion of women with children obtaining an abortion <7 weeks and 7–9 weeks gestation in the prelaw period, but a difference among women at <7 weeks gestation prelaw (50%) versus postlaw (45%, *p*<0.006), suggesting that factors other than the gestational limit may have driven the shift toward fewer women with previous pregnancies (and existing children) obtaining medication abortion in the postlaw period.

## Discussion

We found that a 2011 Ohio law requiring use of an outdated protocol for medication abortion appeared to have disproportionate effects on the most disadvantaged groups. The law seemed to affect younger women, black women, less educated women, and women with Medicaid or no health insurance most. The shift in the sociodemographic profile of medication abortion patients before and after the law is potentially driven by a combination of the lower gestational limit at which medication abortion was available and the increased logistical, financial, time, and travel burdens that the law exacted on women. Women were required to make four visits to the facilities instead of three, resulting in more transportation costs, time away from work and school, and increased need for childcare.

We were able to separate parts of the law that were associated with the declines in use among different sociodemographic subgroups. Both the reduced gestational limit and additional factors, such as logistical and financial burdens, appear to have contributed to the declines among black women and women with less education. The reduced gestational limit for medication abortion (and not the increased burdens) was associated with the decline among younger women, who are known to recognize their pregnancies later,^41^ and Medicaid recipient groups. Finally, the additional logistical and financial burdens imposed by the law (and not the decline in gestational limit) were associated with the decline in medication abortion among women with no insurance and women with children.

Laws requiring use of the FDA-approved protocol do not exist in isolation; they are present in states such as Ohio and Texas, which have many other abortion restrictions in place (e.g., Medicaid coverage restrictions, physician-only ultrasound laws, two-visit requirements, waiting periods).^42^ The cumulative burden of these many requirements may make the service logistically impossible for some women and providers. For others, the additional requirements may cause delays in care, resulting in women seeking abortions at later gestations. These women may have fewer options, becoming ineligible for medication abortion and pushed into later, more costly abortion procedures. While laws like the FDA-protocol restriction may not appear to cause undue burden on their own, their effects must be considered in conjunction with all restrictions on abortion present in a state.

We had only individual-level data on the population who obtained medication abortions. We did not have any data on the population who obtained aspiration or did not obtain an abortion at all. Therefore, we were unable to determine whether women who wanted but did not have a medication abortion in the postlaw period subsequently had an aspiration abortion or did not have an abortion at all. Indeed, research done at abortion clinics throughout Texas in 2014 which has a similar law found women whose nearest clinic had closed were more likely to have a “frustrated demand for medication abortion”—meaning that they reported a preference for medication abortion but ended up having or expecting to have an aspiration abortion—compared with women whose nearest clinic remained open.^43^

This study has a few limitations: This study was observational, relying on pre-/postlaw data with no control group of abortion patients who were not exposed to the law. Thus, causal relationships cannot definitely be drawn. However, the changes in the patterns of patients match exactly with the timing of the implementation of the law. In addition, data used in this study were abstracted from medical charts, allowing the potential for bias. We were unable to blind the abstractors to whether the chart was from the prelaw or postlaw period because the dates of visits were clearly in the charts. In an effort to reduce bias, each abstractor was given extensive training aimed at minimizing bias. In addition, we were lacking data on income, and used insurance coverage and zip code as proxies, limiting our ability to make conclusions on the effects of Ohio's law by income level.

Women have their own personal reasons for preferring medication abortion or aspiration abortion. Offering women a real choice between the two is a component of patient-centered care. State laws on specific types of abortion procedures limit women's ability to access the best and most acceptable healthcare for their situation. State-level restrictions do not affect all groups of women equally and can exacerbate inequities in access to abortion.

## Abbreviations Used

BMI: body mass index
FDA: Food and Drug Administration
GED: general educational development
ITOP: Induced Termination of Pregnancy
LMP: last menstrual period
N.S.: not statistically significant
